# Tunnel/Pouch versus Coronally Advanced Flap Combined with a Connective Tissue Graft for the Treatment of Maxillary Gingival Recessions: Four-Year Follow-Up of a Randomized Controlled Trial

**DOI:** 10.3390/jcm9082641

**Published:** 2020-08-14

**Authors:** Souheil Salem, Leila Salhi, Laurence Seidel, Geoffrey Lecloux, Eric Rompen, France Lambert

**Affiliations:** 1 Department of Periodontology and Oral Surgery, Faculty of Medicine, University of Liège, 4000 Liege, Belgium; souhsal@yahoo.fr (S.S.); l.salhi@chuliege.be (L.S.); geoffrey.lecloux@chuliege.be (G.L.); erompen@hotmail.be (E.R.); 2 Biostatistics and Medico-Economic Information, University Hospital of Liège, 4000 Liège, Belgium; laurence.seidel@chuliege.be

**Keywords:** coronally advanced flap, tunnel technique, connective tissue graft, Miller class 1, gingival recession, root coverage, periodontal plastic surgery

## Abstract

Background: The long-term stability after soft tissue graft for covering gingival recession remains a pivotal goal for both patient and periodontist. Therefore, the aim of this study was to compare the four-year outcomes of the coronally advanced flap (CAF) versus the pouch/tunnel (POT) technique, both combined with connective tissue graft (CTG), for gingival recession treatment. Methods: Forty patients were initially randomly assigned to the control group (CAF + CTG; N = 20) and the test group (POT + CTG; N = 20). Clinical outcomes included mean root coverage (MRC) and complete root coverage (CRC), gingival thickness (GT), and keratinized tissue (KT) gain. Esthetic outcomes were also analyzed using the pink esthetic score (PES) and patient-reported outcome measures (PROMs). All outcomes initially assessed at six months were extended to four years post-surgery. Results: No significant differences were observed between the two patient groups in terms of MRC and CRC. At four years, significantly greater GT and KT gain were noted in the POT + CTG group, and tissue texture enhancement was also more prominent in the test group. Conclusions: The POT + CTG technique allows for long-term clinical coverage of gingival recessions comparable to that of the CAF + CTG technique, but it potentially improves gingival thickness, keratinized tissue and esthetic results.

## 1. Introduction

Gingival recession (GR) is defined as an exposure of the root surface of the tooth resulting from an apical migration of the gingival margin beyond the cemento-enamel junction [1,2,3]. GR is often associated with dentin hypersensitivity, plaque retention and gingival inflammation [4,5]. Moreover, in the anterior region, the esthetic appearance may be compromised and become a major concern for some patients [6].

Over the last decades, several techniques have been proposed for GR treatment, including free gingival grafts [7] and repositioned flaps. Coronally or laterally advanced flaps were widely described alone or in combination with a connective tissue graft (CTG) [8,9,10,11,12,13,14]. According to systematic reviews and meta-analysis [15,16,17,18], coronally advanced flap (CAF) combined with CTG is the most effective technique to treat a single or a multiple recession, and both European Federation of Periodontology(EFP) and American Association of Periodontology(AAP) consensus conferences concluded that this approach should be considered as the gold-standard, whereas conjunction of Enamel Matrix Derivative (EMD) or acellular dermal matrix graft may be considered as alternatives [10,11]. Indeed, according to two recent systematic reviews, mean root coverage (MRC) of 84.7% and 80.9% and complete root coverage (CRC) of 51.8% and 46.6%, respectively, can be expected when using the CAF + CTG approach for Miller’s Class I and II recessions treatment [11,19]. Moreover, it was demonstrated that the adjunction of CTG provides more predictable outcomes (CRC) after a period of five years with less risk of recession relapse [20]. Moreover, a recent 20-year follow up report concluded that the CAF + CTG allowed for stable recession coverage and a gain in long-term keratinized tissue (KT) [20].

Alternative envelope or tunnel techniques combined with CTG were proposed in order to avoid releasing and papilla incisions, and to some extent, decrease the surgical trauma [21,22]. The envelope technique was described for the first time in the literature by Raetzke [23] for single tooth recessions and was later updated for multiple recession coverage [21]. Several authors improved this technique to make it more predictable and less traumatic [22,24,25,26]. The main objective of these different modifications was to achieve better root coverage and optimal soft tissue esthetics [10,14].

The efficacy of the pouch/tunnel technique was recently systematically reviewed, and the authors reported MRC and CRC, reaching 82.75% and 47.15%, respectively, for the treatment of localized recessions [27]. Several randomized controlled trials compared the efficacy of the pouch/tunnel (POT) technique and the CAF + CTG. The results were inconsistent since three Randomized Controlled Trials (RCTs) found no statistical differences between the two techniques, one was in favor of the CAF + CTG, and the other one reported more effective results with the tunnel approach [28,29,30,31,32]. However, to the best of our knowledge, there is only limited data available with a follow-up of more than 12 months [33] and longer controlled trials are needed to verify the clinical benefits.

The first report of the present randomized controlled trial concluded that both surgical techniques are relevant in treating Miller’s class I recession, while the pouch technique seemed to increase the height of KT better and to provide satisfactory gingival-related esthetic outcomes [30]. The present post-hoc study was conducted in order to assess the reliability and stability of outcomes after a period of four years. The primary objective was to compare the four-year outcomes of the CAF and pouch/tunnel (POT) techniques combined with CTG. Secondary objectives concerned keratinized gingiva gain, gingival thickness and esthetic outcomes including the pink esthetic score (PES), as well as patient-reported outcome measures (PROMs).

## 2. Materials and Methods

### 2.1. Study Design

**Figure 1:**
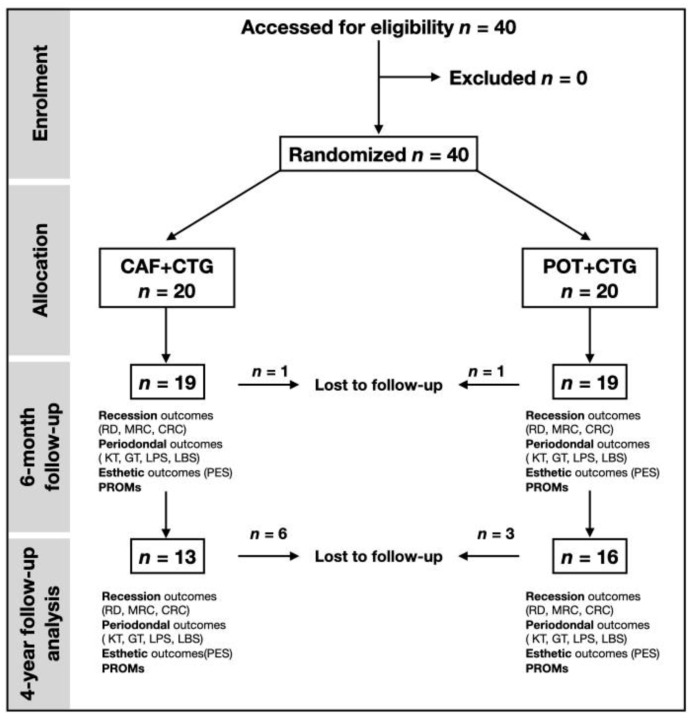
Study design.

This study was based on extended material of a previously reported randomized controlled [30] trial which enrolled a total of 40 patients from the Department of Periodontology and Oral Surgery at the University of Liege, Belgium, requiring treatment of gingival recessions for esthetics and/or dental hypersensitivity. Each patient (experimental unit) contributed to a single recession. In cases of multiple recessions, all the recessions were treated, but only the deepest one was included. Four periodontists were involved in the surgical procedures. A single examiner (L.S.) collected the clinical data and performed the outcome measurements. All clinical parameters and outcomes were evaluated at baseline, after six months, and for the present study, patients were recalled after four years (Figure 1). The RCT was initially powered (80%) to detect a minimal clinically significant difference in root coverage of 1 mm at the 5% significance level. The recession depth (RD) at baseline was considered as a covariate. The four investigators attended two calibration meetings, where the objectives of the study, the surgical protocol, and the assessment method were reviewed.

### 2.2. Study Population

The inclusion criteria were:

1. Miller’s class I recessions;
2. Recession of 2 mm to 5 mm;
3. Maxillary incisors, canines or premolars;
4. Identifiable cementoenamel junction (CEJ);
5. Patients were at least 18 years old;
6. No/controlled periodontal disease;
7. ASA1 or ASA2 (American Society of Anesthesiologists) general health status;
8. Providing a signed informed consent form.

Exclusion criteria were:

1. Smokers;
2. Presence of cervical carious lesion;
3. Pocket depth greater than 4 mm;
4. Sites where previous muco-gingival therapy was performed;
5. Pregnancy.

In the presence of non-carious cervical lesions, the anatomical CEJ was reconstructed by the use of a composite before the procedure.

### 2.3. Patient Inclusion (Informed Consent, Patient Registration and Randomization)

After explaining the purpose, risks, benefits and monitoring of the study, patients were invited to sign an informed consent form. This study was approved by the ethics committee of the University Hospital of Liege (file number: B707201110982) and was registered on ClinicalTrials.gov (NCT04016493).

Patients were subjected to a full periodontal examination. A pre-surgical full-mouth professional prophylaxis appointment was scheduled one week prior to the surgical procedure. An alginate impression was taken to produce an individual resin stent that was used as a reference point for all measurements. After CTG harvesting, the patients were randomly allocated to the CAF + CTG (control group) or POT + CTG (test group) technique. Randomization was performed by lottery through sealed envelopes.

### 2.4. Surgical Procedure

**Figure 2:**
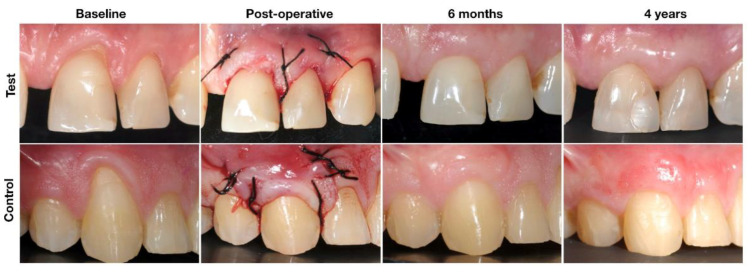
Surgical procedures and follow-up in Test (POT + CTG) and Control (CAF + CTG).

The patient received 600 mg of ibuprofen (paracetamol 1 g in case of allergy) prior to surgery, and chlorhexidine mouthwash 0.2% was provided for 2 min. Patients received local anesthesia at the donor and recession sites (articaine hydrochloride 7200 mg/1.8 mL, adrenalin 1800 mcg/1.8 mL). CTG harvesting was performed prior to the preparation of the reception site to avoid any bias. The graft dimension was calculated according to the recession dimensions; a minimum of 3 mm of the graft was submerged mesially, distally and apically. The CTG was harvested from the palate with single edge incision and sutured with 4.0 silk. The surgical protocol in the control group was performed, as described by Langer and Langer [12]. A horizontal incision at the level of the CEJ and two vertical incisions were designed to raise a split thickness flap beyond the muco-gingival line (MGL). The papillae were desepithelialized. The root was planned using a curette, and a chemical treatment was administered using a doxycycline solution (1 mg/mL) considering its non-antibiotic effects [34,35,36]. The CTG was sutured to the recipient bed with a resorbable suture (Vycril 5.0: Saint Stevens-Woluwe, Belgium), and the flap was coronally advanced and sutured by simple interrupted sutures (Silk 5.0; Ethicon, Johnson and Johnson Company). The connective tissue graft was completely covered by the flap. In the test group, the surgical protocol was performed as described by Raetzke [23] and Allen [21]. The sulcular epithelium was removed with a blade, and a partial thickness pouch was created, preserving the papillae. The roots were treated similarly to those of the control group. Then, the CTG was inserted inside the pouch and stabilized mesially and distally with simple interrupted sutures (silk 5.0), leaving the connective tissue that covered the recession exposed (Figure 2).

Baseline, surgical procedures and sutures placement, six-months follow-up and four years follow-up.

### 2.5. Post-Operative Instructions and Follow-Up

The patients were asked to take painkillers only if necessary and to count the amount of pain killer intake every day for one week. Patients were informed to avoid brushing at the surgical sites for two weeks, to use mouthwash chlorhexidine 0.2% (Perio-aid, Dentaid Benelux, Houten, The Netherlands) until suture removal, and to consume a soft food diet for one week. Sutures were removed after 10 days, patients were initially seen at three and six months, and lastly at four years for the present study.

### 2.6. Data Collection

A single non blinded examiner performed all measurements. Local plaque score (LPS), local bleeding score, RD, recession width (RW), gingival thickness (GT) and KT height were recorded at baseline, six months and four years. GT was measured using an endo-lime 15 and a stop buccally, 1 mm below the cervical limit. All measurements were performed intra-orally using the individual resin stent. The gain in KT height, the MRC, and the percentage of CRC were calculated at six months and four years. The PES was assessed at baseline, six months and four years, according to the seven parameters described by Fürhauser [37]. To assess the reliability of all measurements, the baseline measurements of the four (10%) first patients were scored twice in an interval of one week. Patient-related esthetic outcomes (PROMs) were also recorded by means of a questionnaire using a visual analog scale (VAS).

### 2.7. Statistical Analysis

Results were expressed as mean and standard deviation (SD) for quantitative variables and as number (percent) for categorical findings. Between groups comparisons were made by the unpaired Student’s *t*-test or the Kruskal–Wallis non-parametric test, while the paired Student’s *t*-test was used for assessing changes between two time points. Statistical calculations were always done on the maximum number available on an “intention-to-treat” basis (including drop-outs). Results were considered significant at the 5% critical level (*p* < 0.05). Calculations were performed using SAS software version 9.4 (SAS Institute, Cary, NC, USA).

## 3. Results

### 3.1. Patient Characteristics

The patient and site-related characteristics at baseline for the control and test groups are displayed in Table 1. Similarly, clinical parameters (RD, KT, GT, CRC, MRC, LPS, and local bleeding percentage) and esthetic features recorded at baseline in the two control groups were perfectly comparable (Table 2). From the 40 patients initially included in the study, two patients dropped out after three months of follow-up (one in each group), and 11 patients were lost to follow-up between six months and four years (seven in the control group and four in the test group) (Figure 1).

### 3.2. Recession Coverage

**Figure 3:**
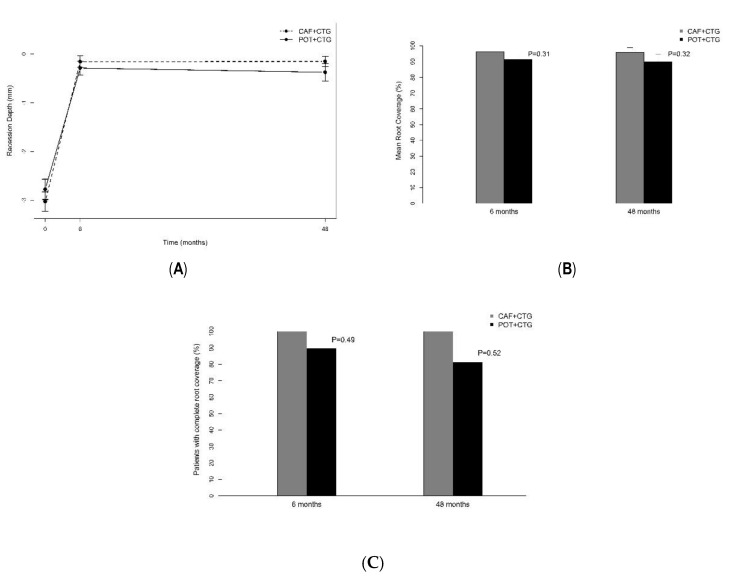
(**A**) Recession depth (mm), (**B**) mean root coverage (%) and (**C**) complete root coverage (%).

From baseline to six months, the mean RD decreased significantly in the two groups (*p* < 0.0001), but thereafter, from six months to four years, it remained unchanged (Figure 3a). The evolutions were perfectly comparable in the two groups (Table 2). At four years the MRC reached 95.9 ± 10.4% in the CAF + CTG group and 90.1 ± 18.2% in the POT + CTG group, and no statistical difference was observed from six months to four years (Figure 3b). Furthermore, the CRC reached 84.6% (11/13) for the CAF + CTG group and 75% (12/16) for the POT + CTG group at 48 months (Figure 3c). Despite a single recurrence observed in the test group, no statistical difference in terms of RD was evidenced from six months to four years in the two groups (*p* = 0.44).

### 3.3. Gain of Keratinized Tissue

As previously observed at six months, a significantly higher gain in KT was found after four years in patients treated by the POT + CTG technique (*p* = 0.0014). Figure S1a explicitly shows that while KT significantly increased from baseline to six months in the POT + CTG group, no change was observed in the CAF + CTG group; thereafter, KT remained unchanged in either group.

### 3.4. Gingival Thickness

The gingival thickness increased significantly and similarly in the two groups (*p* < 0.001) from baseline to six months; thereafter a further augmentation of GT was found in the test group (*p* = 0.007) contrary to the CAF + CTG group (Figure S1b). At four years, the gingiva was significantly thicker in the test group (POT + CTG) (*p* = 0.0012).

### 3.5. Pink Esthetic Score

From baseline to six months, the PES raised significantly in the two groups with better results in the POT + CTG group compared to the CAF + CTG group at six months (*p* = 0.027) and four years (*p* = 0.011). This finding was mainly due to the texture, which averaged 1.94 ± 0.25 in test patients and 1.15 ± 0.38 in control patients (*p* < 0.0001). The other PES features did not differ between the two groups (Table 3).

### 3.6. Patient-Related Esthetic Outcomes

Test and control patients recognized equally a significant esthetic improvement over the four-year follow-up period, mainly in features like aspect, color, contour of the gum and scars.

## 4. Discussion

The present used extended material from a previous RCT to compare the long-term (four years) clinical outcomes of connective tissue graft application for gingival recession treatment using a pouch/tunnel technique versus a CAF technique. The results showed that both techniques allowed a significant recession reduction and these outcomes remained stable over time in both groups. However, in the POT + CTG group, the gain of KT and PES were significantly better both at six months and four years and the gain in gingival thickness observed at six months was confirmed after four years. In both groups, the esthetic improvement was maintained in a similar way over the entire follow-up period for both techniques.

Long-term reports assessing the tunnel technique for recession coverage are limited [33] although long-term follow-ups are much-needed to evaluate the stability of the clinical outcomes of periodontal plastic surgeries [38]. To the best of the authors’ knowledge, the present report is the first RCT-based comparing POT + CTG and CAF + CTG techniques for such a long follow-up period.

### 4.1. Mean Root Coverage (MRC)

The MRCs after a follow-up period of four years were statistically similar and rather high in the two groups (95.9% and 90.1%, respectively). In a recent systematic review evaluating long-term outcomes (≥24 m) for the treatment of recessions, MRC varied from 88.8% to 98.9% with the CAF + CTG technique, while the CAF alone displayed more variable results (from 71.8% to 97.1%) [11]. The authors concluded that the use of a CAF + CTG was the most predictable technique for the long-term stability of root coverage procedure [11]. In another systematic review based on 51 randomized controlled trials, although only a limited number of studies showed long-term outcomes of recession coverage procedure after at least five years of follow-up, better stability and less recurrence were also found with the CAF + CTG technique [10]. On the contrary, over a follow-up period of at least 48 months, the CAF technique without the adjunction of a CTG exhibited a certain degree of recession recurrence [20,39,40,41], while the advanced flaps combined with a CTG displayed better stability over time [42,43,44]. Harris [45] even reported that the MRC tended to improve with time when the CAF + CTG technique was used, based on a study of 100 patients followed for more than two years. However, over a 20 years follow-up period, one study showed that the MRC achieved one year after surgery seemed to slightly decrease overtime independently of the type of the GR [20]. The present results suggest excellent stability in the test and control group. Therefore, it seems that the adjunction of the CTG to limit the risk of recession recurrence is of great importance, as already suggested by some authors [20,43,46]. Moreover, in a recent systematic review and meta-analysis [18], the authors concluded that CAF + CTG maintain long-term stability of root coverage procedures and result in better root coverage outcomes than CAF alone. Nevertheless, recession recurrences seem also to be influenced by other predicting factors, such as the presence of non-carious cervical lesions and tooth brushing habits [10,44].

A few randomized controlled trials compared the efficacy of the CAF + CTG versus POT + CTG after a limited follow-up period of six months or one year [28,29,30,32]; these authors described similar efficacy in MRC with the two techniques, with MRC varying from 80 to 97.3% for the tunnel technique. Only one study reported less effective MRC with the tunnel technique (77.4%) [31]. This inconsistency might be related to surgical skills and strict inclusion criteria. The present study emphasized for the first time that the gain in MRC obtained with the POT technique, over a period of four years, remained as stable as with the gold-standard technique (CAF + CTG). The present RCT considered only Miller Class I recessions and nonsmokers; therefore, the comparisons should be interpreted cautiously despite the fact that the operators were skilled periodontists using well-standardized surgical procedures. Finally, a recent systematic review [27] found an MRC of 83.08% in localized maxillary gingival recession treatment when using the POT + CTG technique. However, it has to be emphasized that only POT with a complete coverage of the CTG were included in the review, while the present study reports the original technique described by Raetzke and Allen [21,23], in which the CTG remains partially exposed.

### 4.2. Complete Root Coverage (CRC)

The present study displayed good stability of the CRC results for up to four years in both groups. These long-term results are comparable to those described in other articles considering the treatment of Miller Class I recessions with the CAF + CTG technique [10,11]. The low rate of recession recurrence found in the present study after four years with the POT + CTG technique is also in accordance with a retrospective study evaluating the envelope technique after 11.4 years of follow-up [33]. On the contrary, in the studies reporting on long-term outcomes of CAF alone, without the adjunction of a CTG, the percentage of sites with CRC decreases significantly overtime [20,40]. Therefore, the use of CTG seems to be of capital importance in order to minimize the recession recurrence irrespectively of the chosen technique (CAF or POT), and these two techniques showed long-term stability in terms of root coverage.

### 4.3. Keratinized Tissue (KT) and Gingival Thickness (GT)

According to a recent systematic review, the gain of KT following root coverage procedures varies from 0.4 to 4.7 mm depending on the techniques applied [11]. Based on long-term studies (≥24 months), the highest gain of KT was found with envelope technique [33] and the lowest with CAF + resorbable membranes [47]. In the present study, the increase of KT and its stability from six months to four years was observed in both groups; however, the augmentation was significantly higher in the POT + CTG compared to the CAF + CTG group. Therefore, according to the present results, the POT + CTG technique may be considered as a technique of choice when the existing KT is limited. Looking at the stability of the regenerated KT using the CAF + CTG technique, our results, although limited to four years, seem to be in accordance with other long-term studies, which concluded that KT improvements achieved by CAF + CTG may be preserved over periods of 10 to 20 years [20,42]. However, as for the tunnel/envelope technique, data on KT gain and stability are scarce in the available literature. Only a 20-year follow-up case series based on 20 patients treated with the envelope technique [33] was found, and the authors described a mean gain in KT of 4.7 mm that was stable over the follow-up period. This KT gain value is much higher than what was found in the present study—however, the difference should be interpreted cautiously as the study design was very different. A significant gain in GT was observed with the two techniques; however, over time, an additional gain was observed in the test group, while it remained stable in the control group. In a recent systematic review on the root coverage procedures, the authors emphasized that in cases where both root coverage and gain in the width of keratinized tissue are expected, the use of subepithelial connective tissue grafts shows a slight improvement in outcome [16]. The reason for this continuous increase of soft tissue thickness in the test group remains unexplained, but it could be related to the fact the graft is exposed and not covered by the mucosal flap, and thus, may behave differently. To the authors’ knowledge, the literature concerning the increase of GT after CTG application in recession coverage treatment is rather limited. Rebele et al. [48] used an intra oral scanner (IOS) to assess the increase of GT from baseline to one year comparing CAF + EMD and POT, and they did not find any difference between the two groups. Nowadays, IOS seems to be a reliable tool for assessing soft tissue profile and volumetric changes [49].

### 4.4. Esthetic Outcomes

Esthetic outcomes were evaluated from both the patient’s and the professional’s points of view. For the esthetic assessment by the professionals, the PES index was chosen [37] as the previous report on the present clinical trial used that specific index [30]. This represents a limitation as the PES was developed for implant esthetics, while the root coverage esthetic score (RES) described by Cairo et al. seems to be considered as a more reliable index for root coverage procedures [50]. The present results indicate that both techniques allowed for a significant improvement of the esthetics, however, better esthetic scores were found in the POT + CTG group both at six months and four years and the main difference was related to the texture values, as already suggested by some authors reporting better esthetic outcomes with a tunnel technique [21,22,24,26]. Indeed, residual scars from the releasing incisions were sometimes still observed after four years. An important aspect of optimizing the esthetic results with the tunnel technique leaving the CTG slightly exposed is the removal of the intrasulcular and junctional epithelium. The importance of this was already emphasized by Raetzke et al. [23]; if the epithelium is left in place, the former gingival margin may remain visible and unaesthetic. Finally, from a patient’s perspective, the two techniques allowed for excellent esthetic results. Patient-centered outcomes related to the esthetics showed significantly improved results from baseline to 48 months although the assessment revealed no statistically significant differences between the groups after 48 months in terms of scars, contours, aspect and color.

### 4.5. Additional Limitations

The present study is a post-hoc analysis of a previously randomized controlled trial designed and conducted by our group. In particular, the RCT was not powered for a four-year comparative assessment of control and test patient groups. Thus, outcome results have to be appraised and interpreted with some caution. Not all initially enrolled patients could be assessed after four years because of drop-outs. Nonetheless, the number of patients remained satisfactory, and data were analyzed on all longitudinal data available whether from complete patients or drop-outs. Finally, the examiner of the present was not blinded, which represents an additional limitation of the present study.

## 5. Conclusions

Both the tunnel and coronally advanced flap techniques combined with a CTG allowed for a significant recession reduction, and clinical outcomes remained stable over a follow-up period of four years in both groups. The tunnel procedure allowed long term stability of increase of keratinized tissues and gain of gingival thickness. Over time, both techniques presented patient esthetic satisfaction—however, the esthetic outcomes evaluated by the dentists were more in favor of the pouch/tunnel technique.

## Figures and Tables

**Table 1:** Patient demographics and site-related characteristics at baseline in control and test groups.

	CAF + CTG *N* = 20	POT + CTG *N* = 20
Gender (male/female)	8/12	6/14
Age (years: mean ± SD)	44.3 ± 13.6	42.8 ± 12.8
No. of teeth treated	27	23
Ratio teeth/patient	5.4	4.6
Single recession site	15	16
Multiple recession site	5	4
Type of teeth		
Incisor	7	5
Canine	18	13
Premolar	2	5

CAF + CTG, coronally advanced flap + connective tissue graft; POT + CTG, Pouch/tunnel + connective tissue graft.

**Table 2:** Comparative evolution of periodontal parameters from baseline to four years in the treated (POT + CTG) and control (CAF + CTG) groups.

Parameter	Group	Baseline Mean ± SD	Six Months Mean ± SD	Four Years Mean ± SD	*p*-Value Baseline vs. Six Months *	*p*-Value Six Months vs. Four Years **
RD (mm)	POT + CTG	2.78 ± 0.94	0.29 ± 0.61	0.38 ± 0.72	<0.0001	0.48
	CAF + CTG	3.03 ± 0.90	0.16 ± 0.50	0.15 ± 0.38	<0.0001	0.67
	*p*-value	0.39	0.47	0.33	0.080	0.44
GT (mm)	POT + CTG	1.08 ± 0.34	1.37 ± 0.37	2.06 ± 0.77	0.0075	0.0075
	CAF + CTG	1.03 ± 0.26	1.25 ± 0.35	1.23 ± 0.33	0.035	1.00
	*p*-value	0.60	0.32	0.0012	0.79	0.019
KT (mm)	POT + CTG	3.55 ± 0.95	4.68 ± 1.06	5.00 ± 0.82	0.0004	0.42
	CAF + CTG	3.28 ± 0.97	3.53 ± 1.12	3.62 ± 1.26	0.52	0.19
	*p*-value	0.37	0.0024	0.0014	0.019	0.88
MRC (%)	POT + CTG	NA	91.3 ± 17.6	90.1 ± 18.2	NA	0.56
	CAF + CTG	NA	96.3 ± 12.1	95.9 ± 10.4	NA	0.79
	*p*-value	NA	0.31	0.32	NA	0.59
CRC (%)	POT + CTG	NA	17/19 (89.5)	13/16 (81.3)	NA	0.32
	CAF + CTG	NA	19/19 (100.0)	13/13 (100.0)	NA	0.99
	*p*-value	NA	0.49	0.52	NA	0.57
LPS (%)	POT + CTG	0.00 ± 0.00	0.00 ± 0.00	1.56 ± 6.3	0.99	0.33
	CAF + CTG	0.00 ± 0.00	0.00 ± 0.00	0.00 ± 0.00	0.99	0.99
	*p*-value	0.99	0.99	0.38	0.99	0.38
LBS (%)	POT + CTG	0.05 ± 0.22	1.32 ± 5.74	1.56 ± 6.25	0.35	0.33
	CAF + CTG	1.25 ± 5.59	1.32 ± 5.74	0.00 ± 0.00	1.00	0.99
	*p*-value	0.34	0.99	0.38	0.59	0.38

* The third *p*-value compares the Baseline–six months differences between the two groups. ** The third *p*-value compares the six months–four years of differences between the two groups. POT + CTG, Pouch technique + connective tissue graft; CAF + CTG, Coronally advanced flap + connective tissue graft RD, recession depth; GT, gingival thickness; KT, keratinized tissue; MRC, mean root coverage; CRC, complete root coverage; LPS, local plaque score; LBS, local bleeding score; NA not applicable.

**Table 3:** Comparative evolution of the esthetic parameters from baseline to four years in the treated (POT + CTG) and control (CAF + CTG) groups.

Parameter	Group	Baseline Mean ± SD	Six Months Mean ± SD	Four Years Mean ± SD	*p*-Value Baseline vs. Six Months *	*p*-Value Six Months vs. Four Years **
Mesial papilla	POT + CTG	1.70 ± 0.47	1.84 ± 0.37	1.88 ± 0.34	0.33	0.33
	CAF + CTG	1.80 ± 0.41	1.84 ± 0.37	1.77 ± 0.44	0.33	0.34
	*p*-value	0.48	0.99	0.47	0.66	0.17
Distal papilla	POT + CTG	1.75 ± 0.44	1.84 ± 0.37	1.94 ± 0.25	0.16	0.33
	CAF + CTG	1.85 ± 0.49	1.89 ± 0.32	1.85 ± 0.38	0.67	0.34
	*p*-value	0.50	0.64	0.44	0.71	0.17
Level of soft tissue margin	POT + CTG	0.95 ± 0.39	2.00 ± 0.00	2.00 ± 0.00	<0.0001	0.99
	CAF + CTG	0.90 ± 0.45	2.00 ± 0.00	1.85 ± 0.38	<0.0001	0.17
	*p*-value	0.71	0.99	0.11	0.42	0.11
Soft tissue contour	POT + CTG	1.30 ± 0.47	1.95 ± 0.23	1.81 ± 0.54	<0.0001	0.33
	CAF + CTG	1.20 ± 0.41	1.89 ± 0.32	1.77 ± 0.44	<0.0001	0.34
	*p*-value	0.48	0.56	0.82	0.77	0.76
Alveolar process	POT + CTG	0.00 ± 0.00	0.00 ± 0.00	0.00 ± 0.00	0.99	0.99
	CAF + CTG	0.00 ± 0.00	0.00 ± 0.00	0.00 ± 0.00	0.99	0.99
	*p*-value	0.99	0.99	0.99	0.99	0.99
Color	POT + CTG	1.85 ± 0.37	2.00 ± 0.00	2.00 ± 0.00	0.083	0.99
	CAF + CTG	1.85 ± 0.37	2.00 ± 0.00	1.92 ± 0.28	0.083	0.34
	*p*-value	0.99	0.99	0.28	0.99	0.28
Texture	POT + CTG	1.15 ± 0.37	1.95 ± 0.23	1.94 ± 0.25	<0.0001	0.99
	CAF + CTG	1.05 ± 0.22	1.32 ± 0.48	1.15 ± 0.38	0.021	0.99
	*p*-value	0.30	<0.0001	<0.0001	0.0007	0.99
PES	POT + CTG	8.70 ± 1.53	11.6 ± 0.61	11.6 ± 0.73	<0.0001	1.00
	CAF + CTG	8.55 ± 1.19	11.0 ± 1.03	10.3 ± 1.65	<0.0001	0.25
	*p*-value	0.73	0.027	0.011	0.44	0.25

* The third *p*-value compares the Baseline–six months differences between the two groups. ** The third *p*-value compares the six months–four years of differences between the two groups. POT + CTG, Pouch technique + connective tissue graft; CAF + CTG, coronally advanced flap + connective tissue graft; PES, Pink Esthetic Score.

## Supplementary Materials

The following are available online at https://www.mdpi.com/2077-0383/9/8/2641/s1, Figure S1a: Height of keratinized tissue, Figure S1b: Gingival thickness.
