# Supplementary figures and images for: Tunnel/Pouch versus Coronally Advanced Flap Combined with a Connective Tissue Graft for the Treatment of Maxillary Gingival Recessions: Four-Year Follow-Up of a Randomized Controlled Trial
Source: J Clin Med. 2020 Aug 14;9(8):2641. doi: 10.3390/jcm9082641 (PMC7466088; doi:10.3390/jcm9082641)

Supplementary file

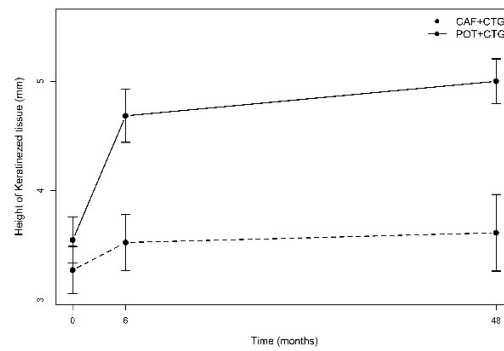

Figure S1 a. Height of keratinized tissue.

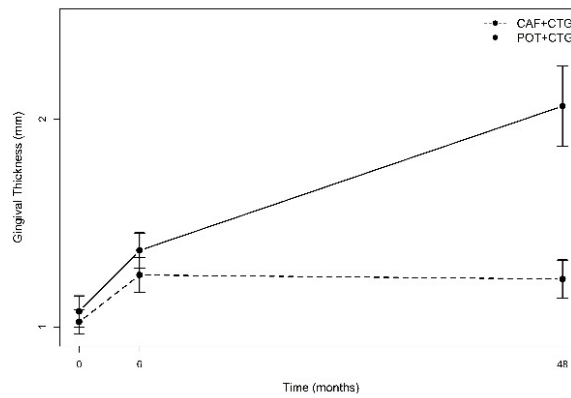

Figure S1 b. Gingival thickness.

Supplement: Supplementary file 1 [file jcm-09-02641-s001.pdf]
